# Suitability of vaccinia virus and bovine viral diarrhea virus (BVDV) for determining activities of three commonly-used alcohol-based hand rubs against enveloped viruses

**DOI:** 10.1186/1471-2334-7-5

**Published:** 2007-02-09

**Authors:** Günter Kampf, Jochen Steinmann, Holger Rabenau

**Affiliations:** 1 Bode Chemie GmbH & Co. KG, Scientific Affairs, Melanchthonstr. 27, 22525 Hamburg, Germany; 2 Institut für Hygiene und Umweltmedizin, Ernst-Moritz-Arndt Universität Greifswald, Walther-Rathenau-Str. 49a, 17489 Greifswald, Germany; 3 MikroLab GmbH, Norderoog 2, 28259 Bremen, Germany; 4 Institut für Medizinische Virologie, Klinikum der Johann Wolfgang Goethe-Universität Frankfurt, Paul-Ehrlich-Str. 40, 60596 Frankfurt, Germany

## Abstract

**Background:**

A procedure for including activity against enveloped viruses in the post-contamination treatment of hands has been recommended, but so far no European standard is available to implement it. In 2004, the German Robert Koch-Institute (RKI) and the German Association for the Control of Virus Disease (DVV) suggested that vaccinia virus and bovine viral diarrhea virus (BVDV) should be used as test viruses in a quantitative suspension test to determine the activity of a disinfectant against all enveloped viruses.

**Methods:**

We have studied the activities of three commonly-used alcohol-based hand rubs (hand rub A, based on 45% propan-2-ol, 30% propan-1-ol and 0.2% mecetronium etilsulfate; hand rub B, based on 80% ethanol; hand rub C, based on 95% ethanol) against vaccinia virus and BVDV, and in addition against four other clinically relevant enveloped viruses: herpes simplex virus (HSV) types 1 and 2, and human and avian influenza A virus. The hand rubs were challenged with different organic loads at exposure time of 15, 30 and 60 s. According to the guidelines of both BGA/RKI and DVV, and EN 14476:2005, the reduction of infectivity of each test virus was measured on appropriate cell lines using a quantitative suspension test.

**Results:**

All three alcohol-based hand rubs reduced the infectivity of vaccinia virus and BVDV by ≥ 4 log_10_-steps within 15 s, irrespective of the type of organic load. Similar reductions of infectivity were seen against the other four enveloped viruses within 15 s in the presence of different types of organic load.

**Conclusion:**

Commonly used alcohol-based hand rubs with a total alcohol concentration ≥ 75% can be assumed to be active against clinically relevant enveloped viruses if they effectively reduce the infectivities of vaccinia virus and BVDV in a quantitative suspension test.

## Background

During the past few years, many healthcare workers have changed from antimicrobial liquid soaps to alcohol-based hand rubs for the post-contamination treatment of hands, as suggested by the CDC guideline for hand hygiene [1]. The two main reasons are broader and faster efficacy, which is beneficial in preventing cross-transmission of nosocomial pathogens [2,3], and superior dermal tolerance, which is beneficial for compliance with hand hygiene regulations [3,4]. Recently it has been suggested that a preparation for routine hand hygiene should be active at least against bacteria, yeasts and enveloped viruses [3]. This minimum spectrum of activity is based on data on the contamination of healthcare workers' hands, on the transmissibility of nosocomial pathogens, and on outbreaks of nosocomial infections caused by contaminated hands of healthcare workers [3]. Quantitative suspension tests are used to determine the spectrum of activity [5]. Recommendations for use in the hospital are usually derived from efficacy tests under practical conditions [5]. Bactericidal activity can be determined according to prEN 13727 (formerly prEN 12054) [6]. It has been shown using a propanol-based hand rub that the four test bacteria of this test norm cover the whole spectrum of nosocomial bacterial strains and clinical isolates including emerging pathogens [7]. For fungicidal activity, only a basic test (EN 1275) is available [8]. One of the two test fungi for EN 1275 is *Candida albicans*, which may serve as the clinically relevant test strain for determining activity against yeasts. To determine virucidal activity, EN 14476:2005 should be used [9]. However, this European norm is only designed for determining "complete virucidal activity", which for hand disinfection includes only two non-enveloped viruses: adenovirus type 5 and poliovirus type 1. No European norm is available for determining activity specific against enveloped viruses, although a national test method has recently been published by the German Association for the Control of Virus Disease (DVV) and the Robert Koch-Institute (RKI) [10]. Such a test norm at the European level would certainly be desirable. The RKI has recently used the phrase "limited virucidal activity" ("active against enveloped viruses such as HIV, HBV, and HCV") to describe a preparation that has proven active against two representative enveloped viruses, vaccinia virus and bovine viral diarrhea virus (BVDV) [11]. BVDV serves as a model for Hepatitis C Virus (HCV) because this pestivirus also belongs to the family Flaviviridae and has some properties similar to HCV [12-14].

To date, however, there is no evidence to show that the two chosen test viruses cover other clinically relevant enveloped viruses in respect of stability against disinfectants. That is why we have tested the activities of three different alcohol-based hand rubs against the two suggested test viruses, and in addition against four other enveloped viruses that represent some of the most relevant or recently-emerging enveloped viruses in human medicine.

## Methods

### Test preparations

The following alcohol-based hand rubs were tested: Preparation A, based on isopropanol (45%, w/w), n-propanol (30%, w/w) and mecetronium etilsulfate (0.2%, w/w); preparation B, based on ethanol (80%, w/w); and preparation C, based on ethanol (95%, w/w). All hand rubs were manufactured by Bode Chemie GmbH & Co. KG, Hamburg, Germany.

### Test viruses

Virus suspensions that allowed a reduction of at least 4 log_10_-steps to be measured were used in all experiments. Infectivity assays were performed between 2003 and 2006 according to the test method of the Bundesgesundheitsamt (BGA, Federal Office of Health, now Robert Koch-Institute) and the German Association for the Control of Virus Disease (DVV) [15], which uses the same test principle as EN 14476, with the following test viruses:

• Vaccinia virus strain Elstree, passaged and cultured in buffalo green monkey cells (BGM cells) (range of virus titres: 7.6–9.9 log_10_TCID_50_/ml)

• Bovine viral diarrhea virus (BVDV) strain NADL, ATCC VR-534, passaged and cultured in KOP-R cells (range of virus titres: 5.5–6.4 log_10_TCID_50_/ml)

• Herpes simplex virus (HSV) type 1 MacIntyre, ATCC VR-539, passaged and cultured in Vero cells (African green monkey kidney, ATCC CCL-81) (range of virus titres: 7.5–8.1 log_10_TCID_50_/ml)

• Herpes simplex virus type 2, ATCC VR-540, passaged and cultured in Vero cells (range of virus titres: 7.6–8.5 log_10_TCID_50_/ml)

• Human influenza A virus, Panama strain 2007/99 (H3N2), passaged and cultured in Madin-Darby canine kidney epithelial cells (range of virus titres: 5.5–5.9 log_10_TCID_50_/ml)

• Avian influenza A virus/duck/Ukraine/1/63 (H3N8), passaged and cultured in Madin-Darby canine kidney epithelial cells (range of virus titres: 6.5–6.9 log_10_TCID_50_/ml). This virus was used as surrogate for H5N1 owing to biosafety considerations.

### Inactivation assay

Tests were conducted in accordance with BGA/RKI and DVV guidelines in a waterbath at 20°C [15]. Eight parts by volume of the disinfectant were mixed with one part by volume of the virus suspension and one part by volume of aqua bidest. This test mixture was investigated at exposure times of 15, 30 and 60 seconds. In tests with different organic loads, one part by volume of the interfering substance was added instead of aqua bidest. The disinfectant was inactivated immediately at the end of the chosen exposure time by serial dilution with ice-cold cell culture medium or by gel filtration with MicroSpin™ S-400 HR columns (Amersham Biosciences Europe GmbH, 79021 Freiburg, Germany), which were used according to the manufacturer's instructions, to reduce the cytotoxicity of the product when BVDV or either of the influenza viruses was tested. Virus controls without columns were run in parallel.

Owing to the addition of virus suspension and organic load, disinfectants could only be evaluated as 80% solutions.

Virus controls were incorporated after the longest exposure time (60 s). One part by volume of virus suspension was mixed with nine parts by volume of aqua bidest. or with one part by volume of organic load and eight parts by volume of aqua bidest.

One experiment was performed for each hand rub, each exposure time, each virus and each type of organic load.

### Types of organic load

Different types of organic load were used (final concentrations):

▪ No organic load (aqua bidest.)

▪10% fetal calf serum

▪ 0.2% bovine serum albumin

▪ 0.03% bovine serum albumin ("clean conditions" according to EN 14476:2005)

▪ 0.3% bovine serum albumin with 0.3% washed sheep erythrocytes ("dirty conditions" according to EN 14476:2005).

### Determination of cytotoxicity

To determine the cytotoxicity of the disinfectants, two parts by volume of aqua bidest. were mixed with eight parts by volume of the disinfectant, diluted with ice-cold cell culture medium and inoculated into permissive cells. Controls for the different organic loads consisted of one part by volume of aqua bidest, one part by volume of organic load and eight parts by volume of the disinfectant. Any microscopic changes in the cells were recorded when the tests were read for cytopathic effects (CPE). This control allowed cytotoxicity and viral CPE to be clearly differentiated.

### Determination of infectivity

Infectivity was determined in a micro-procedure by end-point dilution titration. At the end of each exposure time, the test mixture was immediately diluted with ice-cold cell culture medium, and 100 μl of each dilution were placed in 8 wells of a sterile polystyrene flat-bottomed 96-well microtitre plate (Nunc A/S, 4000 Roskilde, Denmark) with a preformed cell culture monolayer. The microtitre plates were incubated at 37°C with 5% CO_2 _for the appropriate incubation time (3–10 days). Cultures were observed for the presence or absence of CPE. The infective dose (TCID_50_) was calculated according to the method of Spearman [16] and Kärber [17]. Titre reduction was calculated as the difference between the virus titres of the water control (60 s) and the products after the contact times, and is presented as the reduction factor (RF). The following formula was used:

RF = a - b

a = log_10 _TCID_50_/ml of the control titration

b = log_10 _TCID_50_/ml of the test virus titration (after hand rub exposure)

A reduction of infectivity of ≥ 4 log_10_-steps (inactivation ≥ 99.99%) was regarded as evidence of sufficient virucidal activity against the tested virus according German and European guidelines [15].

## Results

The cytotoxicity of the hand rubs was 2 log_10_-steps in all cell lines. Only hand rub C was toxic to MDCK cells at 1:1000 dilution.

All three alcohol-based hand rubs reduced the infectivity of vaccinia virus and BVDV by ≥ 4 log_10_-steps within 15 s, irrespective of the type of organic load (Table 1). Similar reduction factors were seen within 15 s against the other four enveloped viruses (HSV type 1 and 2; human and avian influenza A virus) with or without organic load (Table 1).

**Table 1:** Reduction of viral infectivity (log_10 _steps) obtained with three alcohol-based hand rubs (A: based on 45% isopropanol, 30% n-propanol and 0.2% mecetronium etilsulfate; B: based on 80% ethanol; C: based on 95% ethanol) against the six different enveloped viruses with different types of organic load.

Hand rub	Type of organic load*	BVDV			Vaccinia virus			HSV 1			HSV 2			Human influenza A virus			Avian influenza A virus		
		
		reduction of viral infectivity (log_10_-steps) within a defined contact time**																	
		
		15 s	30 s	60 s	15 s	30 s	60 s	15 s	30 s	60 s	15 s	30 s	60 s	15 s	30 s	60 s	15 s	30 s	60 s
A	None	≥ 4.3			≥ 6.3			≥ 4.0			≥ 4.8			≥ 4.3			≥ 5.1		
	10% FCS	≥ 4.3			≥ 5.3			≥ 4.3			≥ 4.8			≥ 4.4			≥ 5.3		
	0.2% BSA	≥ 4.8			≥ 5.6			≥ 4.0			≥ 5.0			≥ 4.3			≥ 5.3		
	"clean conditions"	≥ 4.7			≥ 5.7			≥ 4.4			≥ 4.1			≥ 4.4			≥ 5.0		
	"dirty conditions"	≥ 4.5			≥ 6.4			≥ 4.4			≥ 4.1			≥ 4.0			≥ 5.4		

B	None	≥ 4.3			≥ 5.0			≥ 4.4			≥ 4.4			≥ 4.3			≥ 5.1		
	10% FCS	≥ 4.0			≥ 5.3			≥ 4.4			≥ 4.4			≥ 4.4			≥ 5.3		
	0.2% BSA	≥ 4.0			≥ 5.3			≥ 4.4			≥ 4.4			≥ 4.3			≥ 5.3		
	"clean conditions"	≥ 4.0			≥ 5.0			≥ 4.6			≥ 4.5			≥ 4.4			≥ 5.0		
	"dirty conditions"	≥ 4.2			≥ 5.4			≥ 4.5			≥ 4.6			≥ 4.0			≥ 5.4		

C	None	≥ 4.4			≥ 4.8			≥ 4.3			≥ 4.4			≥ 4.3			≥ 5.1		
	10% FCS	≥ 4.5			≥ 4.5			≥ 4.3			≥ 4.4			≥ 4.4			≥ 5.3		
	0.2% BSA	≥ 4.9			≥ 4.1			≥ 4.0			≥ 4.4			≥ 4.3			≥ 5.3		
	"clean conditions"	≥ 4.9			≥ 4.5			≥ 4.4			≥ 4.0			≥ 4.4			≥ 5.0		
	"dirty conditions"	≥ 4.7			≥ 5.5			≥ 4.0			≥ 4.4			≥ 4.0			≥ 5.4		

*FCS: 10% fetal calf serum; BSA: 0.2% bovine serum albumin; "clean conditions": (0.03% bovine serum albumin); "dirty conditions": (0.3% bovine serum albumin and 0.3% sheep erythrocytes) **one result is presented in which the log_10 _reduction was the same at all three exposure times

## Discussion

Limited data have been published on the spectrum of virucidal activity of alcohol-based hand rubs [18,19]. In particular, HBV has been described as an enveloped virus that may be less easy to inactivate [19,20].

By testing three commonly-used alcohol-based hand rubs containing at least 75% alcohol, we were able to show that the data obtained with vaccinia virus and BVDV indicated that the preparations are also active against other enveloped viruses relevant to human medicine. According to the declaration of the RKI and DVV the preparations have "limited virucidal activity" and can be considered active against all enveloped viruses relevant to human medicine. "Activity" is the common term in Europe to describe the spectrum of activity in quantitative suspension tests ("in vitro"), whereas "efficacy" is used to describe the effect measured in experiments under practical conditions ("in vivo"). Suspension tests are important for determining the spectrum of activity. Nevertheless, better recommendations can be derived from experiments under practical conditions (phase 2, step 2 tests) such as the ASTM method E-1838-02 [21]. However, some of the above-mentioned viruses cannot be tested on human hands for reasons of biosafety. Furthermore, the type of organic load might strongly influence the virucidal efficacy on hands [22].

All hand rubs were tested at exposure times of 15, 30 and 60 s. The 30 s exposure time best resembles the practical use of hand rubs in hygienic hand disinfection. All the hand rubs tested have a total alcohol concentration of 75% or more. It would also have been interesting to study alcohol-based hand rubs with a lower alcohol concentration to verify the data in a more challenging experimental setting. However, hand rubs with 70% alcohol or less have been shown to have only limited bactericidal activity and should therefore not be used in hospitals [23,24]. Thus, it did not seem to be clinically relevant to verify the activity of such hand rubs against enveloped viruses.

We also found that all the types of organic loads tested in these experiments hardly impaired the activities of the hand rubs against the different enveloped viruses. It has previously been shown using feline calicivirus that the type of organic load may significantly influence the mean reduction of viral infectivity in a test under practical conditions [22]. That is why it seemed crucial to confirm activity not only against different viral species but also under various types of organic load. The real bioburden on healthcare workers' hands, however, may consist of blood, pus, serum, sebum, triglycerides, surface active agents and carbohydrates [25]. The greatest reduction of activity has been reported with blood [25]. It has also been found that the bactericidal efficacy of alcohol-based hand rubs is not significantly impaired if hands are contaminated with 1.2 ml blood [26]. On the basis of our data and the results from Larson and Bobo [24], it is unlikely that the presence of blood would significantly impair the activity of alcohol-based hand rubs against enveloped viruses. For clinical practice it is recommended to use alcohol-based hand rubs if hands are not visibly soiled [1]. Whenever hands are visibly dirty or visibly soiled a hand wash should be performed [1]. That is why our results with rather low quantities of organic load support the confidence into the activity of the tested hand rubs against enveloped viruses when the hand rubs are used as recommended on clean hands.

Viral infectivity was reduced to below the limit of detection in all experiments at 15 s (which is half as long as the current clinical application time for hand rubs) irrespective of the viral species, the type and amount of organic load, and the type of hand rub. It is technically almost impossible to evaluate application times shorter than 15 s in such a test because the disinfectant activity is terminated by immediate dilution. Moreover, it would have only limited clinical relevance, since a single application of an alcohol-based hand rub usually ensures a contact time of 25–30 s before the alcohol is evaporated [24].

Activity against both test viruses in a quantitative suspension test does of course not include the inactivation of non-enveloped viruses in vitro. Entero-, rota-, adeno- and noroviruses e.g. are in general more resistant than BVDV and vaccinia virus. A virucidal activity (effectiveness against all viruses) of a hand disinfectant is difficult to achieve requiring normally a high concentration of ethanol [3] or some kind of synergistic effect. In many situations in medical settings an activity against enveloped viruses is sufficient for a hand rub [3]. On wards with recurrent viral infections or during outbreaks caused by non-enveloped viruses, a hand disinfectant with virucidal activity is absolutely necessary to interrupt the transmission of viruses by human hands.

## Conclusion

Overall, commonly-used alcohol-based hand rubs with 75% alcohol or more can be assumed to be active against clinically relevant enveloped viruses if they reduce the infectivities of vaccinia virus and BVDV in the presence of different standard organic loads by ≥ 4 log_10_-steps. It will be important to confirm our results with selected enveloped viruses including surrogate viruses applying test methods using the finger pads or the whole hand of volunteers.

## Competing interests

The first author is paid employee of Bode Chemie GmbH & Co. KG, Hamburg, Germany, which funded the study.

## Authors' contributions

GK, JS and HR participated in the study design and coordination. JS carried out the experiments. GK drafted the manuscript. All authors read and approved the final manuscript.

## Pre-publication history

The pre-publication history for this paper can be accessed here:


